# Vaccines for Respiratory Viruses—COVID and Beyond

**DOI:** 10.3390/vaccines12080936

**Published:** 2024-08-22

**Authors:** Kalpana Rajanala, Arun Kumar Upadhyay

**Affiliations:** Ocugen Inc., 11 Great Valley Parkway, Malvern, PA 19355, USA; kalpana.rajanala@ocugen.com

**Keywords:** SARS-CoV-2, influenza virus, respiratory syncytial virus, vaccine development, acute respiratory infections

## Abstract

The COVID-19 (coronavirus disease 2019) pandemic had an extensive impact on global morbidity and mortality. Several other common respiratory viruses, such as the influenza virus and respiratory syncytial virus (RSV), are endemic or epidemic agents causing acute respiratory infections that are easily transmissible and pose a significant threat to communities due to efficient person-to-person transmission. These viruses can undergo antigenic variation through genetic mutations, resulting in the emergence of novel strains or variants, thereby diminishing the effectiveness of current vaccines, and necessitating ongoing monitoring and adjustment of vaccine antigens. As the virus-specific immunity is maintained only for several weeks or months after the infection, there is an emergent need to develop effective and durable vaccines. Additionally, specific populations, such as elderly or immunocompromised individuals, may exhibit reduced immune responses to respiratory viruses, posing significant challenges to develop vaccines that elicit durable and potent immunity. We present a comprehensive review of the molecular mechanisms underlying the pathogenesis and virulence of common respiratory viruses, such as RSV, influenza virus, and severe acute respiratory syndrome coronavirus 2 (SARS-CoV-2). We discuss several vaccine approaches that are under development. A thorough understanding of the current strategies and the challenges encountered during the vaccine development process can lead to the advancement of effective next-generation vaccines.

## 1. Introduction

Seasonal respiratory viral infections cause recurring epidemics globally and continue to remain a substantial personal healthcare and economic burden. Several factors, such as antigenic drift, antigenic shift, and species jumps, pose significant challenges in developing vaccines and treatment options. Antigenic drift involves gradual changes in the virus’s surface proteins, leading to the emergence of novel viral variants. The unpredictable nature of antigenic shift, particularly influenza, where the influenza virus acquires new HA (hemagglutinin) or NA (neuraminidase) combinations through reassortment, can create entirely new strains with pandemic potential, requiring rapid vaccine development and reformulation [1]. Inter-species transmission, as seen with influenza, where viruses cross from animals to humans, especially those originating from birds (avian influenza) or pigs (swine influenza), leading to novel and potentially pandemic strains, further complicates vaccine development [2].

According to the World Health Organization (WHO), around one billion individuals are infected with the seasonal influenza virus every year, and three to five million people report severe illness. Seasonal influenza is reported to cause 290,000 to 650,000 respiratory deaths annually [3]; among these, 99% of the deaths are in children under five years of age [4]. Acute respiratory viral infection caused by respiratory syncytial virus (RSV) is also emerging as a major cause of global morbidity and mortality and accounts for 2.1 million outpatient visits, as well as 58,000 to 80,000 hospitalizations annually among children younger than five years of age (Centers for Disease Control and Prevention). Severe acute respiratory syndrome coronavirus 2 (SARS-CoV-2) has infected over 700 million individuals and resulted in over 7 million deaths to date [5], and it continues to be highly contagious, leading to a vast number of hospitalizations and deaths due to the emergence of novel variants.

People infected with influenza virus exhibit symptoms, such as fever, cough, sore throat, muscle aches, and fatigue, as a result of the immune response and tissue damage. Severe infection in some instances can lead to pneumonia, acute respiratory distress syndrome (ARDS), and secondary bacterial infections. Symptoms of RSV infection include cough, wheezing, difficult breathing, and fever. Severe cases can result in bronchiolitis and pneumonia, especially in infants, older adults, and immunocompromised individuals. COVID-19 symptoms range from mild respiratory symptoms to severe pneumonia, ARDS, and multi-organ failure. Complications include thrombosis, cytokine storms, and long-term effects, such as “long COVID”. Vaccination is an effective and cost-efficient method to curtail epidemics resulting from respiratory viruses and preserve community health. Influenza viruses have caused four pandemics so far [6] and are widely studied for vaccine development. However, the efficacy of current influenza vaccines varies from 10–60% every year [7,8]. Current vaccines available for SARS-CoV-2 have reduced the frequency of severe illness and mortality, although they fail to provide robust cross-protection against emerging variants and are unable to control transmission and reinfection. To counteract the constantly mutating viral variants, there is an emerging need to develop next-generation effective vaccines against seasonal and pandemic respiratory viruses with robust systemic and mucosal immunity, durability, and ability to prevent infections [9].

In this review, we discuss the pathogenesis and virulence of common respiratory viruses, such as the influenza virus, RSV, and SARS-CoV-2. These viruses present unique challenges due to their ability to mutate, the need for updated vaccines to match circulating strains, and the vulnerability of certain populations, such as young children and adults over 65, to severe illness. We highlight several innovative vaccine approaches, such as mRNA vaccines, attenuated live vaccines, viral vector vaccines, protein subunit vaccines, and DNA vaccines that are under development. We discuss the challenges encountered towards the advancement of these vaccines. Effective vaccines against these respiratory viruses can be delivered by an extensive understanding of the current approaches and by identifying the opportunities to develop universal next-generation vaccines.

## 2. History of Development of Respiratory Viral Vaccines

The 1918–1919 Spanish influenza pandemic affected one-fifth of the world’s population and caused over 50 million deaths and infections worldwide [10]. The first influenza vaccine developed in the 1930s was a chemically inactivated killed-virus vaccine [11]. In the 1940s, Jonas Salk, later famous for the polio vaccine, conducted early work on influenza vaccine development [12]. During the 1950s, researchers began to develop methods for growing influenza viruses in fertilized chicken eggs. This technique is still used in vaccine production today [12]. In the U.S., the first inactivated influenza vaccine was approved in 1945 [13]. In 1976, an inactivated swine flu vaccine was introduced in response to an outbreak scare, which was associated with some adverse events and led to the development of Guillian–Barré Syndrome (GBS) in certain recipients [14]. The advancement of live attenuated influenza vaccines (LAIV) began in the 1970s, leading to the approval of FluMist^TM^ in the early 2000s [15]. Advances in biotechnology allowed for the development of recombinant influenza vaccines. Flublok, the first recombinant influenza vaccine, was approved by the FDA in 2013 [16,17]. The 2009 H1N1 influenza pandemic emphasized the need for more effective and flexible influenza vaccine strategies and prompted global efforts to improve vaccine production capacity and response times. Various types of influenza vaccines now exist, including Fluarix^®^ (Glaxo Smith Kline Biologicals (GSK)), Agriflu^®^ and Fluad^®^ (Novartis), FluLaval^®^ (ID Biomedical Corporation of Quebec), and Afluria^®^ (bioCSL). Research continues into universal influenza vaccines, which aim to provide broader and longer-lasting immunity against constantly mutating virus strains.

Research into RSV began in the 1950s and 1960s after the virus was discovered in the 1950s. The first formalin-inactivated RSV vaccine trials in the 1960s led to the development of adverse events, such as enhanced respiratory disease (ERD), with vaccinated children experiencing severe respiratory illness upon natural exposure to the virus [18]. Efforts to develop an RSV vaccine slowed after the 1960s setback due to safety concerns. Subunit vaccines and live attenuated vaccines were explored, but challenges were faced in achieving sufficient immunity without causing harmful side effects. Advances in molecular biology and vaccine technology led to renewed interest in RSV vaccine development. Arexvy^®^ (GSK) and Abrysvo^®^ (Pfizer) are the recently approved vaccines that are indicated for the prevention of lower respiratory tract infection in the aging population. Abrysvo^®^ (Pfizer) can be additionally used for maternal immunization to confer passive immunity to infants [19]. Research continues into novel vaccine approaches, such as mRNA vaccines and nanoparticle vaccines, with the goal of developing a safe and effective RSV vaccine. The vaccine candidates under clinical trials for RSV are extensively reviewed by Topalidou et al. [19].

The coronavirus outbreak started in December 2019 and spread rapidly to over 185 countries by the end of 2020; it was declared to be a global pandemic. The spike protein of SARS-CoV-2 was identified as the primary target for vaccine development [20,21], and several platforms were explored, including mRNA, viral vector, protein subunit, and inactivated virus vaccines [22]. The Pfizer-BioNTech’s (BNT162b2) [23,24] and Moderna’s (mRNA-1273) [25,26] vaccines, which utilized mRNA technology, were among the first to receive Emergency Use Authorization (EUA) from the U.S. FDA in December 2020. The prompt development and approval of mRNA vaccines played a role in curtailing the spread and reducing the mortality rate significantly [25,26,27]. Meanwhile, AstraZeneca’s viral vector vaccine, Vaxzevria (AZD1222) [28,29] and Johnson & Johnson’s single-dose viral vector vaccine, Janssen/Ad26.COV2.S, also gained authorization in early 2021 [30,31]. Furthermore, Russia’s Sputnik V [32], and CanSino’s Convidecia (Ad5) have been approved for use in several countries [33]. Additionally, inactivated vaccines, such as China’s Sinopharm (using the HB02 virus strain), Sinovac (Coronavac utilizing the CN2 virus strain) [34], as well as India’s Covaxin [35], were developed and used for immunization. The global effort marked by the rapid production and distribution of billions of doses has been crucial in controlling the pandemic and reducing the severity and mortality of COVID-19. Since its outbreak, over 183 vaccines to protect against SARS-CoV-2 have been in clinical development, the majority of which are either RNA-based or protein subunit vaccines [36].

## 3. Molecular Mechanisms Underlying Pathogenesis and Virulence

All three viruses cause respiratory infections by infecting and damaging cells of the respiratory tract. The immune response plays a vital role, both in fighting infection and contributing to tissue damage and inflammation. Severe cases of these viral infections can lead to respiratory failure, pneumonia, and other life-threatening complications. Understanding the molecular pathophysiology of these respiratory viruses is crucial for the development of effective vaccines, treatments, and preventive measures. Research continues to uncover new insights into these mechanisms to improve our ability to combat these infections.

### 3.1. Viral Entry

**Figure 1:**
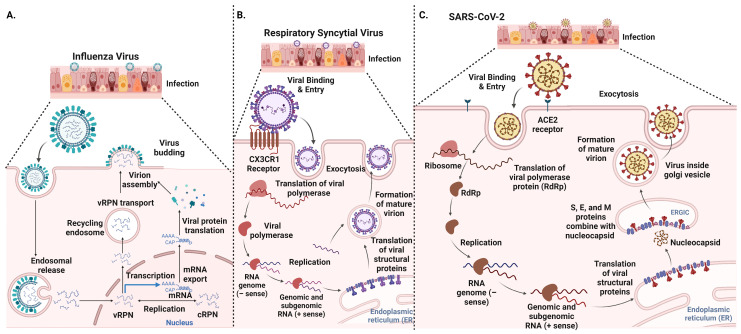
Viral entry and replication mechanisms of common respiratory viruses—(**A**) influenza viruses (**B**), respiratory syncytial virus, and (**C**) SARS-CoV-2.

Influenza viruses primarily infect the respiratory epithelium, including the cells lining the upper respiratory tract (nose, throat) and the lower respiratory tract (lungs). The virus enters host cells through the binding of its surface protein, hemagglutinin (HA), to sialic acid receptors on the host cell membrane [37] (Figure 1A). RSV infects the respiratory epithelial cells of the lungs, causing bronchiolitis and pneumonia, particularly in infants and young children. The virus binds cell surface receptors, including glycosaminoglycans and the CX3CR1 receptor [38,39] (Figure 1B). SARS-CoV-2 invades host cells by binding its spike protein (S protein) to the angiotensin-converting enzyme 2 (ACE2) receptor on the cell surface [40,41] (Figure 1C). Once attached to the host cell surface, the influenza virus enters the cell via endocytosis, which allows it to evade immune surveillance and gain access to the host cell’s cytoplasm (Figure 1A). The other respiratory viruses, such as coronaviruses and RSV, can directly fuse their viral envelope with the host cell membrane, bypassing the endocytic pathway (Figure 1B,C). This process releases the viral genome into the host cell cytoplasm for replication.

Receptor-independent entry mechanisms are often employed influenza, RSV, and SARS-CoV-2 that allow them to exploit the host cell’s machinery and enter via macropinocytosis [42,43,44] and clathrin- or caveolin-mediated endocytosis pathways [45,46,47]. These processes involve the virus being engulfed by the cell membrane and enclosed within vesicles where the acidic environment within facilitates the fusion of the viral envelope with the vesicle membrane, releasing the viral genome into the cytoplasm. Influenza and SARS-CoV-2 gain receptor-independent entry, primarily through pH-dependent endocytosis mechanisms [48]; specifically, a pH-sensitive enzyme, such as cathepsin L, is required by the SARS-CoV-2 during endosomal transport [49]. RSV’s F (fusion) protein facilitates the formation of syncytia, which are multinucleated giant cells, by promoting direct fusion between infected and neighboring uninfected cells. This cell-to-cell fusion allows RSV to propagate within tissues while evading extracellular immune responses, and it is an effective mechanism for local virus spread and evasion of immune detection, which is critical for RSV’s pathogenesis and persistence in the respiratory tract [43]. In addition to the above mechanisms, all the three viruses can also employ lipid rafts, which are specialized membrane microdomains rich in cholesterol and sphingolipids, to facilitate viral internalization [50,51,52].

### 3.2. Replication, Protein Synthesis, and Assembly: Life Cycle of Respiratory Viruses

The replication process and cellular sites of replication for influenza, respiratory syncytial virus (RSV), and SARS-CoV-2 exhibit significant differences due to their unique viral structures and infection mechanisms. Influenza virus primarily replicates in the nucleus of host cells, which is atypical for RNA viruses [53]. After entering the host cell, its viral RNA-dependent RNA polymerase transcribes and replicates viral RNA within the nucleus and directs the synthesis of viral proteins, including hemagglutinin (HA), neuraminidase (NA), and others [54,55]. In contrast, RSV viral RNA is transcribed and replicated by its RNA-dependent RNA polymerase in the cytoplasm using the host cell’s machinery [43]. The RSV viral RNA polymerase replicates the genome and synthesizes viral proteins, which plays a key role in viral entry and cell-to-cell spread [56]. SARS-CoV-2 also replicates in the cytoplasm, but its replication process involves a distinctive compartmentalization within the host cell. After entering the host cell, SARS-CoV-2’s RNA is replicated and transcribed in the cytoplasm, with assembly occurring in the endoplasmic reticulum–Golgi intermediate compartment (ERGIC) [57]. This compartmentalization contrasts with the more straightforward cytoplasmic replication of RSV and the nuclear replication of influenza. SARS-CoV-2 viral proteins, including the spike (S), envelope (E), membrane (M), and nucleocapsid (N) proteins, are synthesized and assembled into new viral particles within the host cell [58]. These differences highlight the diverse strategies employed by these viruses to hijack host cellular machinery and propagate within their respective environments.

### 3.3. Humoral and Cellular Immunity against Respiratory Viruses

**Figure 2:**
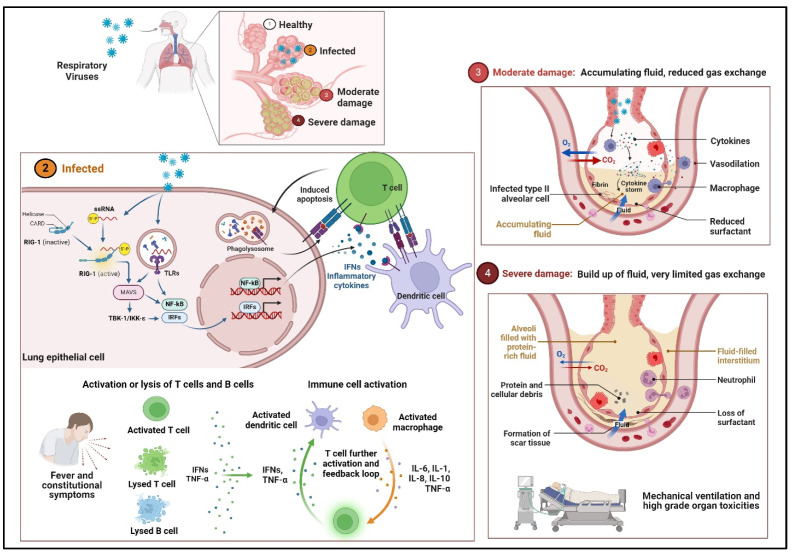
Immune and cellular responses to respiratory viruses—activated RIG-I (retinoic acid-inducible gene I), a pattern recognition receptor in the cytosol forms a secondary structure with the viral RNA and interacts with the adaptor mitochondrial antiviral-signaling protein (MAVS). Immune cells, including macrophages, identify the virus and produce cytokines. The activation of downstream signaling pathways and transcription factors lead to the induction of innate immune responses via the production of inflammatory cytokines. Cytokines attract more immune cells, which in turn cause them to release additional cytokines, creating an inflammation loop that damages the lung cells through the formation of fibrin. Progression to severe damage results from weakened blood vessels, allowing fluid to seep in and fill the lung cavities, leading to respiratory failure. NF-κB: Nuclear factor kappa B; TLR: Toll-like receptor; IRFs: interferon regulatory factors; TNF-α: tumor necrosis factor-alpha; ILs: interleukins.

Once inside the cell, the viral RNA is sensed by the pattern recognition receptors (PRRs), such as retinoic acid-inducible gene 1 (RIG-I) and Toll-like receptors (TLRs) [59] (Figure 2). The specific PRRs used by different respiratory viruses are listed in Table 1. The replication process of the influenza virus damages the cells, resulting in the release of inflammatory cytokines and causing cell death [60]. Proinflammatory cytokines mostly originate from monocytes, macrophages, and neutrophils, which are recruited to and stimulated by chemokines or cytokines, like TNF and IL-6 [61,62]. This positive feedback loop results in the worsening of damage (Figure 2). The immune reaction encompasses the activation of T and B lymphocytes and subsequently triggers antibody production. RSV infection damages the respiratory epithelial cells, leading to inflammation and mucus production [63]. The immune response, which results in cytokine release and the recruitment of immune cells, contributes to tissue damage (Figure 2). The release of cytokines (cytokine storm), can cause acute respiratory distress syndrome, ultimately resulting in tissue damage and organ dysfunction [64,65] (Figure 2).

## 4. Current Vaccine Approaches against Respiratory Viruses

### 4.1. Inactivated Vaccines and Attenuated Live Vaccines

Inactivated viral vaccines are produced by treating the virus with heat, chemicals, or radiation, rendering it unable to replicate or cause disease. Inactivated vaccines are generally considered safe because they do not contain live virus particles, thus making them suitable for use in immunocompromised individuals and pregnant women. Fluviral^®^ or FluLaval™ (GSK), an inactivated split virus vaccine, was marketed for influenza [66]. Development of inactivated vaccine candidates, such as PiCoVacc, Coronavac, Covaxin, and BBIBP-CorV, for SARS-CoV-2 was also reported [67,68]. Inactivated vaccines are stable and do not require refrigeration during storage and transportation. Inactivated vaccines elicit a weaker and shorter duration of immunity compared to live attenuated vaccines or viral vector vaccines. Additional booster doses or adjuvants may be required to enhance and maintain immunogenicity. Inactivated viral vaccines may cause local reactions, such as pain or swelling at the injection site, or mild and transient systemic responses, such as fever or malaise.

Live attenuated vaccines contain weakened forms of the virus that are still able to replicate but cause minimal or no disease in vaccinated individuals [69]. These vaccines can stimulate robust immune responses, including both cellular and humoral immunity, similar to natural infection, leading to long-lasting immunity after a single dose or a few doses [70]. Live attenuated vaccines can mimic natural infection by replicating in the respiratory tract, leading to the induction of mucosal immune responses [71]. In many cases, live attenuated vaccines offer protection with a single dose and are often administered orally or intranasally, eliminating the need for needles and syringes, thus simplifying vaccine administration and improving compliance, especially in children, where multiple doses may be challenging to administer [72]. FluMist^®^ was the first LAIV approved by the FDA and has been widely used, particularly in children and young adults. Developed using the Ann Arbor cold-adapted strain, FluMist^®^ replicates effectively in the cooler environment of the upper respiratory tract, contributing to its attenuation and safety [15]. Other cold-adapted LAIVs, such as Fluenz in Europe [73] and Russian LAIV [74], have also shown good safety profiles and the ability to induce both systemic and mucosal immunity. MEDI ΔM2-2 and RSV LID ΔM2-2 are live attenuated RSV vaccines with a deletion of the M2-2 gene to reduce viral replication while maintaining immunogenicity [75,76]. Despite showing promise in early trials, concerns about mild respiratory symptoms in infants and viral shedding raised safety questions of these vaccines [77]. COVI-VAC, a live attenuated COVID-19 vaccine developed by Codagenix, Inc., uses a synthetic biology platform for “codon deoptimization”, reducing the virus’s replication without altering its antigenic properties [78]. This approach allows the vaccine to mimic natural infection closely, potentially inducing a strong and broad immune response. Early clinical trials have shown that COVI-VAC is safe and induces both humoral and cellular immunity, with the potential for long-lasting protection [79]. MV-014-212, another live attenuated COVID-19 vaccine by Meissa Vaccines, uses an attenuated RSV backbone to express the SARS-CoV-2 spike protein. It has shown promise in early trials for inducing mucosal immunity through intranasal administration, potentially preventing viral transmission [80].

Overall, live attenuated vaccines can induce immunity in vaccinated individuals and confer herd immunity by reducing viral transmission within communities. However, with this vaccination technique, there is a theoretical risk that live attenuated viruses could revert to a virulent form and cause disease in vaccinated individuals. Therefore, these vaccines are generally not recommended for use in immunocompromised individuals, pregnant women, or individuals with severe allergies to vaccine components due to safety concerns [81]. Pre-existing immunity to related viruses may interfere with the replication and effectiveness of live attenuated vaccines. Although rare, live attenuated vaccines can cause vaccine-associated disease in susceptible individuals, such as vaccine-strain viral shedding or mild symptoms resembling the disease being prevented. Live attenuated vaccines may require strict temperature control during storage and transportation to maintain their potency. Despite these disadvantages, live attenuated vaccines are valuable tools for preventing respiratory viral infections, offering strong and long-lasting immunity with relatively few doses.

### 4.2. mRNA Vaccines

RNA molecules are small and encode specific antigens that are highly immunogenic. mRNA vaccines have a very favorable safety profile because they do not integrate into the host’s genome. The antigen production process is very similar to the viral replication in the human cell upon infection. Moreover, the in vitro production process is fast and scalable, with a lesser likelihood of contamination from cell cultures that could potentially alter the antigenicity of the final product [82,83]. Two types of mRNA with varied biological properties are considered for vaccination purposes: self-amplifying mRNA (SAM) and non-replicating mRNA [82].

One of the major disadvantages of using mRNA molecules is that they are fragile and susceptible to physiological degradation (Figure 2). One solution is the use of lipid nanoparticles to encase the molecules into a complex structure to improve the stability of mRNA vaccines [84]. This renders the antigen expression transient and thereby requires high and repeated doses of administration to generate the required efficacy. Another limitation of mRNA vaccines is the loss of efficacy against the new emerging variants of viruses [85]. These vaccines can still be the primary choice for vaccine development during global outbreaks due to the fast and simple manufacturing process. During the coronavirus pandemic, Pfizer-BioNtech’s BNT162b2 [86] and Moderna’s mRNA-1273 [25] were developed rapidly and proved to be highly efficacious. RSV mRNA vaccine, which encodes stabilized F glycoprotein of RSV, mRNA-1345, is under development [87]. Monovalent and quadrivalent mRNA vaccines for influenza are under clinical development [88,89,90], and efforts to develop multivalent mRNA vaccines to protect against different influenza subtypes are underway [91,92]. Adverse effects of mRNA vaccines developed against COVID-19 include anaphylaxis in few cases, occurring within minutes to hours after vaccination [93]. Rare events, such as myocarditis and pericarditis, were also observed, particularly in younger age groups, following mRNA vaccination for COVID-19 [94,95,96,97]. The available research for understanding the biological mechanisms linking mRNA vaccines and myocarditis is still limited.

### 4.3. Protein Subunit Vaccines

Protein subunit vaccines are composed of specific proteins or protein fragments derived from the target pathogen, such as the structural membrane proteins of SARS-CoV-2, such as the S (spike), M (membrane), E (envelope), and N (nucleocapsid) protein [98], F, G, and M proteins of RSV [99] or the core proteins of the influenza virus, such as HA (hemagglutinin), or NA (neuraminidase) [100]. The subunit vaccine candidates developed for SARS-CoV-2 are reviewed and listed in detail by Dong et al. and Heidary et al., and for RSV and the influenza virus they are reviewed by Topalidou et al. and Chen et al., respectively [19,98,100,101]. These vaccines are well-characterized and highly pure and are less likely to be affected by pre-existing immunity against the whole pathogen or related antigens, potentially improving vaccine efficacy. They do not contain live viruses or genetic material from the pathogen, thus minimizing the risk of contaminants or the risk of causing disease or adverse reactions associated with other vaccine types. Protein subunit vaccines elicit specific desired immune responses, such as neutralizing antibodies or cellular immunity against respiratory viruses. These vaccines are typically stable and can be stored at refrigerated temperatures, simplifying storage and distribution logistics compared to vaccines requiring cold-chain storage. Some of the disadvantages of protein subunit vaccines include their lower immunogenicity compared to live attenuated or viral vector vaccines because of the lack of intrinsic adjuvant properties and the structural complexity of whole pathogens [101]. Therefore, protein subunit vaccines need adjuvants or delivery systems to enhance their immunogenicity and may require booster doses to maintain durable immunity (Figure 2). Manufacturing protein subunit vaccines involves several processes, such as protein expression, purification, and formulation, which can be expensive and time-consuming. These limitations affect vaccine accessibility and scalability, especially during pandemics or global outbreaks. Moreover, respiratory viruses, such as influenza viruses, undergo antigenic drift and shift, leading to the emergence of new strains with different antigenic properties [102]. Subunit vaccines may need to be updated regularly to match circulating viral strains, requiring continuous surveillance and production adjustments. Despite these challenges, protein subunit vaccines remain a valuable approach for developing safe and effective vaccines against respiratory viruses, particularly when tailored adjuvants and delivery systems are used to enhance immunogenicity.

### 4.4. DNA Vaccines

DNA vaccines utilize plasmid DNA that encodes viral pathogenic proteins, and these induce cellular and humoral immunity in hosts [103]. The simplicity with which plasmid DNA can be modified to change the nature of the immune response is one of the main benefits of DNA vaccination. The ZyCoV-D vaccine was developed for the coronavirus-containing plasmid DNA encoding signal peptide and SARS-CoV-2 spike protein S genes [104]. Results from the phase 3 trial for the ZyCoV-D vaccine demonstrated that it was immunogenic and safe to use [105]. INO-4800 is another DNA vaccine that encodes SARS-CoV-2 spike S protein [106]. Results from a Phase 1 open-label trial indicated that INO-4800 was well tolerated by all participants [107], and its development as a primary and booster vaccine candidate is underway [108].

The safety concerns for DNA vaccines include the persistence of plasmids in vivo for a prolonged period, the development of autoimmune responses due to the presence of CpG motifs in the plasmid backbone, and the production of cytokines that hamper the vaccine response. As the targeted population for the vaccines, especially for RSV, mainly includes infants and children, their immature immune systems may develop tolerance instead of immunity to the exposed antigens. To overcome these challenges and to improve the immunogenicity of DNA vaccines, coadministration of plasmids encoding for cytokines or costimulatory molecules can be considered [103].

### 4.5. Viral Vector Vaccines

Viral vector vaccines use a modified virus to deliver genetic material encoding antigens of interest to host cells, stimulating an immune response against the target pathogen. Adenoviruses are widely used vectors [109]; others, such as lentivirus, vaccinia virus, and Sendai virus, are also utilized to deliver the antigen [110]. Viral vectors can efficiently deliver antigen-encoding genes to host cells, leading to robust and prolonged cellular and humoral immune responses with a single dose, simplifying vaccine administration and improving compliance. Some viral vector vaccines can be designed to induce mucosal immune responses, providing an additional layer of protection at the site of infection. Viral vector vaccines can be engineered to express different antigens by simply swapping the genetic material encoding the antigen of interest. This flexibility makes viral vector platforms adaptable for the development of vaccines against various respiratory viruses.

**Figure 3:**
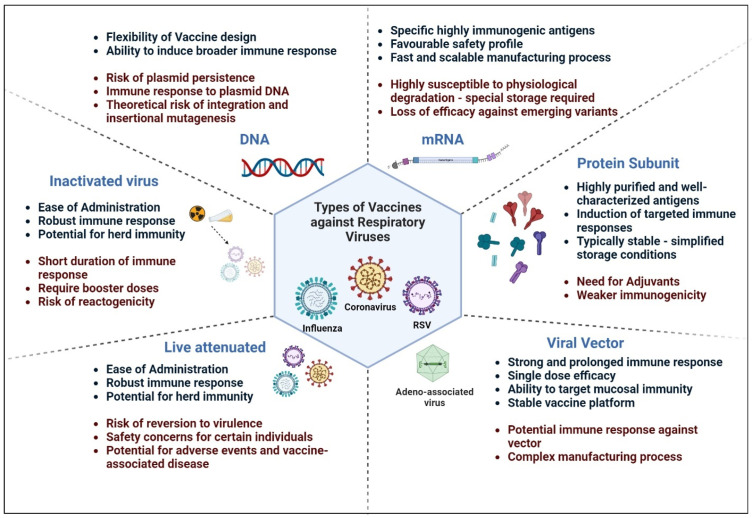
Types of vaccines for respiratory viruses—different kinds of vaccines that were considered or are under development to combat respiratory viruses are illustrated. Their respective advantages (in blue) and disadvantages (in red) are also listed.

The various viral vectors used for vaccine development, such as adenoviruses, adeno-associated viruses (AAV), etc., are extensively reviewed by Travieso et al. [111]. Adenoviral vector-based vaccines are being widely developed for respiratory viruses (Figure 3), leveraging different adenovirus types and vectors to induce strong immune responses. The AdHu5 vector (human adenovirus type 5) has also been explored for influenza vaccines, though pre-existing immunity in the population poses challenges. Similarly, the Ad4-H5-Vtn (human adenovirus type 4) vector has shown potential against avian influenza, demonstrating strong antibody responses [112]. The various adenoviral vector vaccines being developed for influenza are reviewed by Sayedahmed et al. [113]. The modified vaccinia Ankara (MVA) vector, originally a smallpox vaccine, has been adapted to carry influenza virus antigens, such as nucleoprotein (NP) and matrix protein 1 (M1). This vector is highly immunogenic and has been explored as a platform for universal influenza vaccines that could provide protection against multiple strains and subtypes of the flu virus [114]. GSK’s ChAd155-RSV uses a chimpanzee adenovirus vector vaccine expressing three RSV antigens: the F (fusion), N (nucleoprotein), and M2-1 (matrix) proteins. It has been shown to be immunogenic, generating strong antibody and T-cell responses against RSV [115]. Bavarian Nordic’s MVA-BN-RSV employs the MVA vector, showing promise in lowering both symptom scores and viral load in early trials [116]. Janssen manufactured Ad26.RSV.preF, which also showed high efficacy against RSV in older adults [117]. Against SARS-CoV-2, AstraZeneca’s Vaxzevria (AZD1222, based on chimpanzee adenovirus ChAdOx1) [28,29], and Johnson & Johnson’s Janssen/Ad26.COV2.S (human adenovirus 26) vaccines [30,31] have proved to be highly efficacious and are being used globally, with the latter noted for its single-dose efficacy. Russia’s Sputnik V uses a combination of Ad26 and Ad5 vectors, achieving high efficacy [32], while CanSino’s Convidecia (Ad5) has been approved in several countries [33]. Other adenoviral vector vaccine platforms for SARS-CoV-2 are extensively reviewed by Mendonça et al. [118].

The factors that limit the effectiveness of viral vector vaccines are pre-existing immunity or immune responses against the viral vector, potential for vector recombination, insertional mutagenesis, or unexpected adverse events. Although most viral vectors are designed to be non-integrating, there is a theoretical risk of genomic integration of vector DNA into the host genome, which could potentially lead to oncogenesis or other long-term health effects. However, extensive preclinical and clinical testing is typically conducted to assess the safety of viral vector vaccines. Viral vector vaccines often require complex manufacturing steps, including virus propagation, genetic engineering, and purification, often requiring specific storage conditions, such as cold-chain storage, to maintain stability and efficacy. These processes can affect vaccine accessibility and scalability, especially during pandemics.

## 5. Role of Adjuvants for Vaccine Delivery

Advances in delivery systems and research on novel vaccine adjuvants to prime T cell populations and induce more robust and durable immune responses are critical to advance vaccine development. Adjuvants improve targeted antigen presentation by enhancing the uptake of the antigen by antigen-presenting cells (APCs), such as dendritic cells and macrophages, thereby helping the immune system to recognize and respond more effectively to the specific pathogen without reacting to similar, non-target antigens [119]. Adjuvants activate various innate immune pathways, leading to the production of cytokines and chemokines that help shape a more specific adaptive immune response [119,120]. For example, CpG oligodeoxynucleotides mimic microbial DNA, and can stimulate Toll-like receptors (TLRs) on immune cells, enhancing the production of antibodies and the activation of T cells specific to the vaccine antigen [121]. By enhancing the initial immune response, adjuvants also increase the duration of T-cell responses and promote the development of long-lasting immunological memory [122]. This ensures that upon subsequent exposure to the pathogen, the immune system can mount a rapid and effective response, reducing the cross-reactivity with non-target antigens. Some adjuvants help to balance the type of immune response, such as promoting a Th1 or Th2 response depending on the desired outcome [122]. This balance helps prevent the immune system from generating a broad, less specific response that could lead to cross-reactivity. Adjuvants can reduce the amount of antigen needed in a vaccine, which allows for more precise targeting of the immune response, thereby contributing to advantages, such as antigen conservation as with dose sparing [123]. Moreover, adjuvants, like MF59 and AS03, which are oil-in-water emulsions, create an inflammatory environment that promotes the differentiation of B cells into long-lived plasma cells and supports the generation and maintenance of memory B and T cells [124]. These memory cells reside in bone marrow and mucosal-associated lymphoid tissues, providing sustained antibody production and rapid responses upon re-exposure to the pathogen that are critical for long-term immunity, and they can quickly expand upon encountering the antigen again, ensuring durable protection [125]. Overall, adjuvants can contribute to vaccine durability by increasing antibody persistence and the longevity of the immune response.

Aluminum-based adjuvants are the most commonly used vaccine adjuvants; however, they did not enhance the immunogenicity of influenza vaccines [126]. Polysaccharides, glycolipids, liposomes, TLR agonists, nanoparticles, plant-derived amphipathic glycosides or saponins, etc., are being explored as adjuvants for respiratory viral vaccines [127]. Other particulate adjuvants that have similar dimensions to pathogens, such as microparticles, virosomes, emulsions and virus-like particles, can be efficiently taken up by APCs to induce immune responses [119]. Matrix-M, a modified saponin adjuvant used in conjunction with cholesterol or phospholipids, stimulates innate immune receptors, such as TLRs, leading to the activation of immune pathways that promote the production of mucosal IgA antibodies, and was proven to be efficacious in vaccine clinical trials for influenza [128] and COVID-19 [129]. Arexvy, developed by GSK, for RSV utilizes a liposome-based vaccine adjuvant called AS01E which contains QS-21, a saponin-based immune stimulant [130]. A comprehensive list of adjuvants under clinical trials in conjunction with respiratory viral vaccines are listed in Table 2.

## 6. Limitations of Current Vaccines

Egg-based flu vaccines show antigenic drift during production, which reduces vaccine efficacy. Currently licensed vaccines for SARS-CoV-2 demonstrate limited durability, with a waning efficiency of 13.6% per month, a decrease to ~22% from 1 to 5 months post-vaccination, and low booster compliance (<20%) [131,132]. Vaccines that are delivered parenterally via intramuscular injection face some limitations for vaccine roll-out and for protective efficacy. The antigenic drift observed due to the changes occurring over time in the surface proteins, such as hemagglutinin (HA), neuraminidase (NA) of the influenza virus, or the surface proteins of SARS-CoV-2, pose challenges for antigen recognition and development of specific immune responses [1,133]. While the approved COVID-19 vaccines have played a pivotal role in controlling the pandemic, they have some drawbacks. Major concerns include rare instances of myocarditis and pericarditis, which were observed particularly among younger males receiving mRNA vaccines [94,95,96,97]. Additionally, some individuals have experienced allergic reactions, including anaphylaxis, though these were infrequent occurrences [93]. There is also evidence of waning vaccine effectiveness, necessitating booster doses to maintain immunity, especially against emerging variants, like the Delta and Omicron strains [134]. Furthermore, vaccine hesitancy remains a significant challenge due to the misinformation and concerns over their safety and long-term effects, which hinders the efforts to achieve widespread immunity [135]. Manufacturing challenges include adapting vaccines to seasonal variants, as well as increasing production scale and release to meet the demand. The short duration of elicited serum immunity against the constantly mutating respiratory viral vaccines can be combated by the development of combination vaccines.

## 7. Strategies to Improve Vaccine Efficacy for Respiratory Viruses

**Figure 4:**
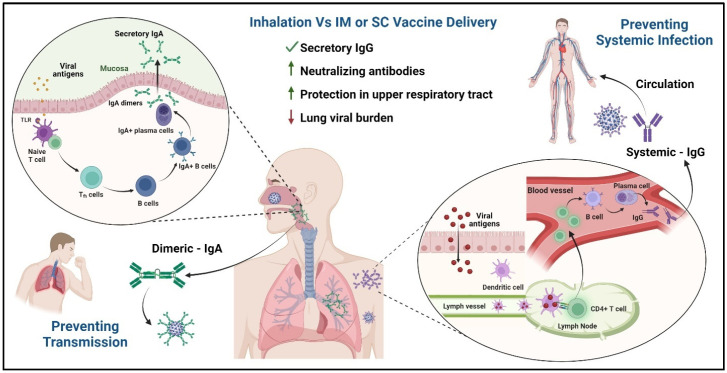
Advantages of the inhalation vaccine platform—the inhalation route generates a mucosal (IgA) response and robust humoral immune response (IgG), thus generating higher titers of neutralizing antibodies. The secretory IgA in the upper respiratory tract is especially helpful in conferring protection, reducing infection burden, and preventing transmission.

The interactions of influenza, RSV, and SARS-CoV-2 with the immune system during the induction and effector phases occur primarily in the respiratory and oral mucosa. The mucosal immune system is the largest and first line of defense against pathogens, with a capacity to neutralize biologically active antigens. It also has advantages over systemic immune responses due to its effector functions, such as the inhibition of antigen uptake and neutralization of viruses inside epithelial cells [136]. The intramuscular or subcutaneous route of delivery induces systemic IgG antibodies and can suppress infection in the lower respiratory tract. However, these methods result in lower protection conferred by IgG antibodies in the upper respiratory tract mucosa. As with natural infection, the mucosal route of immunization generates virus-specific IgA antibodies by the mucosa-associated lymphoid tissues (MALT) in the upper respiratory tract (Figure 4), resulting in mucosal and systemic immunity. In preclinical animal studies performed with Balb/c mice, ACE-2 transgenic mice, hamsters, and rhesus macaques, the intranasal route of vaccination generated a superior immune response to SARS-CoV-2 as compared to intramuscular administration vaccination [137,138,139]. Mucosal immune responses to vaccines and the advantages of next-generation mucosal vaccines are reviewed in detail by Mettelman et al. and Dotiwala et al., respectively [140,141]. The mucosal route of vaccination approach is superior to injectable vaccines in terms of effectiveness and cross-protection against recurrent infection and the prevention of transmission [142].

The challenges in delivering the vaccines via the mucosal route include dilution in mucosal secretions, degradation by proteases and nucleases [143], and the development of immune tolerance due to low uptake of soluble or non-adherent antigens [144]. These challenges can be overcome by utilizing adenoviral vectors as the preferred method to develop mucosal vaccines. Adenoviral vectors were shown to efficiently transduce cells in the mucosal layers of the airways and can induce innate and adaptive immune responses without the need for adjuvants [145,146,147]. The various intranasal/inhalation vaccines that are approved or under clinical trials are listed in Table 3.

## 8. Conclusions and Future Directions

Numerous heterogeneous risk groups and emerging seasonal variants continue to pose major challenges in the development of vaccines against circulating respiratory viruses. While designing next-generation respiratory vaccines, key factors, such as vaccine type, route of administration, boosting strategies, and immunization frequencies, should be carefully considered. A deeper understanding of host immune mechanisms has paved the way for the mucosal route of administration of vaccines, which is proving to be more advantageous than other methods to confer optimal protection and prevent transmission. There are several ongoing trials evaluating potential vaccine candidates that are safe, easy to administer, have wider acceptability amongst the high-risk population, and which can offer a longer duration of protection. However, adverse effects, such as vaccine-induced viral shedding, the development of ERD, and the potential off-target effects impacting other tissues and organs, should be carefully evaluated during the advancement of new vaccines, before licensing them for widespread use.

## Figures and Tables

**Table 1:** Comparison of the description and characteristics of common respiratory viruses.

	Influenza Virus	Respiratory Syncytial Virus	SARS-CoV-2
Family	*Orthomyxoviridae*	*Paramyxoviridae*	*Coronaviridae*
Description	Enveloped viruses with genome comprising of segmented negative-sense single-strand RNA segments	Enveloped, negative-sense, single-stranded RNA virus consisting of 11 proteins encoded by a 15.2-kb RSV genome	Enveloped positive-sense RNA viruses, characterized by club-like spikes projecting from their surface, with a large RNA genome of ~30 kb
Subtypes	Four genera: influenza virus A–D (IAV, IBV, ICV, and IDV)	Members include human RSV, bovine RSV, and murine pneumonia virus. Two major antigenic human RSV (A and B)	Phylogenetic analyses classify four genera: alphacoronaviruses and beta coronaviruses (majorly infecting mammals); gamma coronaviruses and delta coronaviruses (avian)
Viral entry	Hemagglutinin (HA) processing by trypsin-like proteases	The fusion protein (F) mediates the fusion of the virus with the host membrane and releases the nucleocapsid into the host cell cytoplasm	Spike protein processing by host proteases, such as transmembrane serine protease 2 (TMPRSS2), cathepsin L and furin, and neuropilin 1
Host receptor	Sialic acid	chemokine receptor, CX3CR1	ACE2
Viral replication	Nuclear	Cytoplasmic	Cytoplasmic
Viral recognition in epithelial cells	Intracellular receptors, such as TLR3 (Toll-like receptor), RIG-1 (retinoic acid-inducible gene 1), and ZBP1 (Z-DNA binding protein 1)	Intracellular receptors, such as TLR7, TLR8, and RIG-1	Intracellular receptors, such as TLR3, RIG-1 (retinoic acid-inducible gene 1), and MDA5 (melanoma differentiation-associated 5)

**Table 2:** Adjuvants that are approved or under clinical trials for respiratory viral vaccines.

**Influenza Virus**		
**Adjuvant**	**Description**	**Clinical Trial**
LTh(αK)	A heat-labile enterotoxin (LT) derived from *E. coli*	NCT03784885
MF59	Oil-in-water emulsion composed of squalene	NCT00133471
JVRS-100	A cationic liposome–DNA complex	NCT00662272
Matrix-M	A modified saponin adjuvant used in conjunction with cholesterol or phospholipids	NCT04120194
**Respiratory Syncytial Virus**		
**Adjuvant**	**Description**	**Clinical Trial**
AS01E	Liposome-based vaccine adjuvant containing QS-21, a saponin-based immune-stimulant	NCT04841577
**SARS-CoV-2**		
**Adjuvant**	**Description**	**Clinical Trial**
CpG	Cytosine phosphoguanine—a synthetic DNA sequence that activates TLR9 and enhances innate and adaptive immune responses	NCT05385991
LTh(αK)	A heat-labile enterotoxin (LT) derived from *E. coli*	NCT05069610

**Table 3:** Intranasal or inhalation vaccines that are approved or under trials for influenza, RSV, and COVID-19.

**Influenza Virus**				
**Vaccine**	**Manufacturer**	**Type**	**Mucosal Version**	**Clinical Trial Status**
FluMist^TM^	AstraZeneca	Live attenuated	Intranasal	Approved (USA)
NASOVAC-S4	Serum Institute of India	Live attenuated	Intranasal	Approved (India)
M2 Deleted Single Replication (M2SR) H3N2 influenza vaccine	FluGen Inc.	Live attenuated	Intranasal	Phase 1 Trial (NCT05163847)
DNS1-RBD (influenza)	Beijing Wantai BioPharm (China)	Viral vector	Intranasal	In Phase 2 trial
CyanVac (CVXGA1-001) parainfluenza	CyanVac (USA)	Viral vector vaccine	Intranasal	In Phase 2 trial (NCT04954287)
**Respiratory Syncytial Virus**				
**Vaccine**	**Manufacturer**	**Type**	**Mucosal Version**	**Clinical Trial Status**
RSV DNS2/D1313/ I1314L, RSV 6120/ DNS2/1030s, RSV 276	National Institute of Allergy and Infectious Diseases (NIAID)	Live attenuated	Intranasal	Phase 1/2 trial (NCT03916185)
RSV vaccine formulation 1/2	Sanofi Pasteur	Live attenuated	Intranasal	Phase 2 trial (NCT04491877)
MV-012-968	Meissa Vaccines	Live attenuated	Intranasal	Phase 1/2 trial (NCT04690335)
BLB-201	Blue Lake Biotechnology	Viral vector (parainfluenza virus 5)	Intranasal	Phase 1/2 trial (NCT05655182)
**SARS-CoV-2**				
**Vaccine**	**Manufacturer**	**Type**	**Mucosal Version**	**Clinical Trial Status**
iNCOVACC (BBV154)	Bharat Biotech	Viral vector	Intranasal	Approved (India)
Convidecia Air (Ad5-nCoV)	CanSino (China)	Viral vector (adenovirus)	Inhaled through the mouth using a nebulizer	Authorized in China, Sep 2022
Pneucolin	Beijing Wantai BioPharm	Viral vector vaccine	Intranasal	Authorized in China, Dec 2022
GAM-COVID-VAC (rAd26-S—Sputnik Light)	Gamaleya Research Institute (Russia)	Viral vector (adenovirus)	Intranasal	Authorized in Russia, April 2022
Ad5-triCoV/Mac & ChAd-triCoV/Mac	Canadian Institutes of Health Research (Canada)	Viral vector (adenovirus)	Aerosol	In Phase 1 Trial (NCT05094609)
SC-Ad6-1	Moat Biotechnology Corporation	Viral vector (adenovirus)	Intranasal or inhaled	In Phase 1 Trial (NCT04839042)
CoviLiv	Codagenix (USA)	Live attenuated	Intranasal spray	In Phase 3 (NCT04619628)
ACM-001	ACM Biolabs (Singapore)	Protein subunit	Intranasal	In Phase 1 Trial (NCT05385991)
Mambisa	Center for Genetic Engineering and Biotechnology (Cuba)	Protein subunit (nucleocapsid)	Intranasal drops	Completed Phase 2
Avacc 10	Novotech (Australia)	Protein Subunit	Intranasal	Phase 1 Trial (NCT05604690)
B/HPIV3/S-6P	National Institute of Allergy and Infectious Diseases	Recombinant Live attenuated Viral vector	Intranasal (nasal spray)	Phase 1 Trial (NCT06026514)

## Abbreviations

COVID-19—Coronavirus disease 2019; RSV—respiratory syncytial virus; SARS—severe acute respiratory syndrome; CoV-2—Coronavirus 2; RSV—respiratory syncytial virus; WHO—World Health Organization; ARDS—acute respiratory distress syndrome; ERD—enhanced respiratory disease; mRNA—messenger ribonucleic acid; HA—hemagglutinin; CX3CR1—chemokine (C-X3-C) receptor 1; ERGIC—endoplasmic reticulum–Golgi intermediate compartment; ACE2—angiotensin-converting enzyme 2; LAIV—live attenuated influenza vaccine; GBS—Guillian–Barré Syndrome; ERD—enhanced respiratory disease; TLR—Toll-like receptor; RIG-1—retinoic acid-inducible gene I; MDA5—melanoma differentiation-associated 5; NF-κB—nuclear factor kappa B; ILs—interleukins; IRFs—interferon regulatory factors; Ig—immunoglobulin; PRRs—pattern recognition receptors; DNA—deoxyribonucleic acid; NA—neuraminidase; TNF-α—tumor necrosis factor-alpha; MALT—mucosa-associated lymphoid tissues; APCs—antigen-presenting cells.

## References

[1] Petrova V.N., Russell C.A. (2018). The evolution of seasonal influenza viruses. Nat. Rev. Microbiol..

[2] Short K.R., Richard M., Verhagen J.H., van Riel D., Schrauwen E.J., van den Brand J.M., Manz B., Bodewes R., Herfst S. (2015). One health, multiple challenges: The inter-species transmission of influenza A virus. One Health.

[3] Iuliano A.D., Roguski K.M., Chang H.H., Muscatello D.J., Palekar R., Tempia S., Cohen C., Gran J.M., Schanzer D., Cowling B.J. (2018). Estimates of global seasonal influenza-associated respiratory mortality: A modelling study. Lancet.

[4] World Health Organization Influenza (Seasonal); Fact Sheet No. 211. https://www.who.int/news-room/fact-sheets/detail/influenza-(seasonal).

[5] Roknuzzaman A., Sarker R., Nazmunnahar, Shahriar M., Mosharrafa R.A., Islam M.R. (2024). The WHO has Declared COVID-19 is No Longer a Pandemic-Level Threat: A Perspective Evaluating Potential Public Health Impacts. Clin. Pathol..

[6] Saunders-Hastings P.R., Krewski D. (2016). Reviewing the History of Pandemic Influenza: Understanding Patterns of Emergence and Transmission. Pathogens.

[7] Uno N., Ross T.M. (2024). Multivalent next generation influenza virus vaccines protect against seasonal and pre-pandemic viruses. Sci. Rep..

[8] Dhakal S., Klein S.L. (2019). Host Factors Impact Vaccine Efficacy: Implications for Seasonal and Universal Influenza Vaccine Programs. J. Virol..

[9] Morens D.M., Taubenberger J.K., Fauci A.S. (2023). Rethinking next-generation vaccines for coronaviruses, influenzaviruses, and other respiratory viruses. Cell Host Microbe.

[10] Stern A.M., Cetron M.S., Markel H. (2010). The 1918-1919 influenza pandemic in the United States: Lessons learned and challenges exposed. Public Health Rep..

[11] Francis T. (1953). Vaccination against influenza. Bull. World Health Organ..

[12] Barberis I., Myles P., Ault S.K., Bragazzi N.L., Martini M. (2016). History and evolution of influenza control through vaccination: From the first monovalent vaccine to universal vaccines. J. Prev. Med. Hyg..

[13] Weir J.P., Gruber M.F. (2016). An overview of the regulation of influenza vaccines in the United States. Influenza Other Respir. Viruses.

[14] Fiore A.E., Bridges C.B., Cox N.J. (2009). Seasonal influenza vaccines. Curr. Top. Microbiol. Immunol..

[15] Carter N.J., Curran M.P. (2011). Live attenuated influenza vaccine (FluMist(R); Fluenz): A review of its use in the prevention of seasonal influenza in children and adults. Drugs.

[16] Baxter R., Patriarca P.A., Ensor K., Izikson R., Goldenthal K.L., Cox M.M. (2011). Evaluation of the safety, reactogenicity and immunogenicity of FluBlok(R) trivalent recombinant baculovirus-expressed hemagglutinin influenza vaccine administered intramuscularly to healthy adults 50-64 years of age. Vaccine.

[17] Cox M.M., Izikson R., Post P., Dunkle L. (2015). Safety, efficacy, and immunogenicity of Flublok in the prevention of seasonal influenza in adults. Ther. Adv. Vaccines.

[18] Mejias A., Rodriguez-Fernandez R., Oliva S., Peeples M.E., Ramilo O. (2020). The journey to a respiratory syncytial virus vaccine. Ann. Allergy Asthma Immunol..

[19] Topalidou X., Kalergis A.M., Papazisis G. (2023). Respiratory Syncytial Virus Vaccines: A Review of the Candidates and the Approved Vaccines. Pathogens.

[20] Lan J., Ge J., Yu J., Shan S., Zhou H., Fan S., Zhang Q., Shi X., Wang Q., Zhang L. (2020). Structure of the SARS-CoV-2 spike receptor-binding domain bound to the ACE2 receptor. Nature.

[21] Zhang J., Xiao T., Cai Y., Chen B. (2021). Structure of SARS-CoV-2 spike protein. Curr. Opin. Virol..

[22] Bayani F., Hashkavaei N.S., Arjmand S., Rezaei S., Uskokovic V., Alijanianzadeh M., Uversky V.N., Ranaei Siadat S.O., Mozaffari-Jovin S., Sefidbakht Y. (2023). An overview of the vaccine platforms to combat COVID-19 with a focus on the subunit vaccines. Prog. Biophys. Mol. Biol..

[23] Thomas S.J., Moreira E.D., Kitchin N., Absalon J., Gurtman A., Lockhart S., Perez J.L., Perez Marc G., Polack F.P., Zerbini C. (2021). Safety and Efficacy of the BNT162b2 mRNA Covid-19 Vaccine through 6 Months. N. Engl. J. Med..

[24] Polack F.P., Thomas S.J., Kitchin N., Absalon J., Gurtman A., Lockhart S., Perez J.L., Perez Marc G., Moreira E.D., Zerbini C. (2020). Safety and Efficacy of the BNT162b2 mRNA Covid-19 Vaccine. N. Engl. J. Med..

[25] Baden L.R., El Sahly H.M., Essink B., Kotloff K., Frey S., Novak R., Diemert D., Spector S.A., Rouphael N., Creech C.B. (2021). Efficacy and Safety of the mRNA-1273 SARS-CoV-2 Vaccine. N. Engl. J. Med..

[26] El Sahly H.M., Baden L.R., Essink B., Doblecki-Lewis S., Martin J.M., Anderson E.J., Campbell T.B., Clark J., Jackson L.A., Fichtenbaum C.J. (2021). Efficacy of the mRNA-1273 SARS-CoV-2 Vaccine at Completion of Blinded Phase. N. Engl. J. Med..

[27] Roest S., Hoek R.A.S., Manintveld O.C. (2021). BNT162b2 mRNA Covid-19 Vaccine in a Nationwide Mass Vaccination Setting. N. Engl. J. Med..

[28] Voysey M., Clemens S.A.C., Madhi S.A., Weckx L.Y., Folegatti P.M., Aley P.K., Angus B., Baillie V.L., Barnabas S.L., Bhorat Q.E. (2021). Safety and efficacy of the ChAdOx1 nCoV-19 vaccine (AZD1222) against SARS-CoV-2: An interim analysis of four randomised controlled trials in Brazil, South Africa, and the UK. Lancet.

[29] Clemens S.A.C., Folegatti P.M., Emary K.R.W., Weckx L.Y., Ratcliff J., Bibi S., De Almeida Mendes A.V., Milan E.P., Pittella A., Schwarzbold A.V. (2021). Efficacy of ChAdOx1 nCoV-19 (AZD1222) vaccine against SARS-CoV-2 lineages circulating in Brazil. Nat. Commun..

[30] Barouch D.H., Stephenson K.E., Sadoff J., Yu J., Chang A., Gebre M., McMahan K., Liu J., Chandrashekar A., Patel S. (2021). Durable Humoral and Cellular Immune Responses 8 Months after Ad26.COV2.S Vaccination. N. Engl. J. Med..

[31] Sadoff J., Gray G., Vandebosch A., Cardenas V., Shukarev G., Grinsztejn B., Goepfert P.A., Truyers C., Fennema H., Spiessens B. (2021). Safety and Efficacy of Single-Dose Ad26.COV2.S Vaccine against Covid-19. N. Engl. J. Med..

[32] Jones I., Roy P. (2021). Sputnik V COVID-19 vaccine candidate appears safe and effective. Lancet.

[33] Halperin S.A., Ye L., MacKinnon-Cameron D., Smith B., Cahn P.E., Ruiz-Palacios G.M., Ikram A., Lanas F., Lourdes Guerrero M., Munoz Navarro S.R. (2022). Final efficacy analysis, interim safety analysis, and immunogenicity of a single dose of recombinant novel coronavirus vaccine (adenovirus type 5 vector) in adults 18 years and older: An international, multicentre, randomised, double-blinded, placebo-controlled phase 3 trial. Lancet.

[34] Hu L., Sun J., Wang Y., Tan D., Cao Z., Gao L., Guan Y., Jia X., Mao J. (2023). A Review of Inactivated COVID-19 Vaccine Development in China: Focusing on Safety and Efficacy in Special Populations. Vaccines.

[35] Sapkal G.N., Yadav P.D., Ella R., Deshpande G.R., Sahay R.R., Gupta N., Vadrevu K.M., Abraham P., Panda S., Bhargava B. (2021). Inactivated COVID-19 vaccine BBV152/COVAXIN effectively neutralizes recently emerged B.1.1.7 variant of SARS-CoV-2. J. Travel. Med..

[36] Zasada A.A., Darlinska A., Wiatrzyk A., Woznica K., Forminska K., Czajka U., Glowka M., Lis K., Gorska P. (2023). COVID-19 Vaccines over Three Years after the Outbreak of the COVID-19 Epidemic. Viruses.

[37] Mair C.M., Ludwig K., Herrmann A., Sieben C. (2014). Receptor binding and pH stability—How influenza A virus hemagglutinin affects host-specific virus infection. Biochim. Biophys. Acta (BBA)-Biomembr..

[38] Johansson C. (2016). Respiratory syncytial virus infection: An innate perspective. F1000Research.

[39] Johnson S.M., McNally B.A., Ioannidis I., Flano E., Teng M.N., Oomens A.G., Walsh E.E., Peeples M.E. (2015). Respiratory Syncytial Virus Uses CX3CR1 as a Receptor on Primary Human Airway Epithelial Cultures. PLoS Pathog..

[40] Flerlage T., Boyd D.F., Meliopoulos V., Thomas P.G., Schultz-Cherry S. (2021). Influenza virus and SARS-CoV-2: Pathogenesis and host responses in the respiratory tract. Nat. Rev. Microbiol..

[41] Li W., Moore M.J., Vasilieva N., Sui J., Wong S.K., Berne M.A., Somasundaran M., Sullivan J.L., Luzuriaga K., Greenough T.C. (2003). Angiotensin-converting enzyme 2 is a functional receptor for the SARS coronavirus. Nature.

[42] Rossman J.S., Leser G.P., Lamb R.A. (2012). Filamentous influenza virus enters cells via macropinocytosis. J. Virol..

[43] Collins P.L., Fearns R., Graham B.S. (2013). Respiratory syncytial virus: Virology, reverse genetics, and pathogenesis of disease. Curr. Top. Microbiol. Immunol..

[44] Zhang Y.Y., Liang R., Wang S.J., Ye Z.W., Wang T.Y., Chen M., Liu J., Na L., Yang Y.L., Yang Y.B. (2022). SARS-CoV-2 hijacks macropinocytosis to facilitate its entry and promote viral spike-mediated cell-to-cell fusion. J. Biol. Chem..

[45] Gutierrez-Ortega A., Sanchez-Hernandez C., Gomez-Garcia B. (2008). Respiratory syncytial virus glycoproteins uptake occurs through clathrin-mediated endocytosis in a human epithelial cell line. Virol. J..

[46] Glebov O.O. (2020). Understanding SARS-CoV-2 endocytosis for COVID-19 drug repurposing. FEBS J..

[47] Lakadamyali M., Rust M.J., Zhuang X. (2004). Endocytosis of influenza viruses. Microbes Infect..

[48] Aganovic A. (2023). pH-dependent endocytosis mechanisms for influenza A and SARS-coronavirus. Front. Microbiol..

[49] Zhao M.M., Yang W.L., Yang F.Y., Zhang L., Huang W.J., Hou W., Fan C.F., Jin R.H., Feng Y.M., Wang Y.C. (2021). Cathepsin L plays a key role in SARS-CoV-2 infection in humans and humanized mice and is a promising target for new drug development. Signal Transduct. Target. Ther..

[50] Verma D.K., Gupta D., Lal S.K. (2018). Host Lipid Rafts Play a Major Role in Binding and Endocytosis of Influenza A Virus. Viruses.

[51] Chang T.H., Segovia J., Sabbah A., Mgbemena V., Bose S. (2012). Cholesterol-rich lipid rafts are required for release of infectious human respiratory syncytial virus particles. Virology.

[52] Palacios-Rápalo S.N., De Jesús-González L.A., Cordero-Rivera C.D., Farfan-Morales C.N., Osuna-Ramos J.F., Martínez-Mier G., Quistián-Galván J., Muñoz-Pérez A., Bernal-Dolores V., Del Ángel R.M. (2021). Cholesterol-Rich Lipid Rafts as Platforms for SARS-CoV-2 Entry. Front. Immunol..

[53] Dou D., Revol R., Ostbye H., Wang H., Daniels R. (2018). Influenza A Virus Cell Entry, Replication, Virion Assembly and Movement. Front. Immunol..

[54] Houser K., Subbarao K. (2015). Influenza vaccines: Challenges and solutions. Cell Host Microbe.

[55] Bergeron H.C., Tripp R.A. (2022). RSV Replication, Transmission, and Disease Are Influenced by the RSV G Protein. Viruses.

[56] Zimmer G., Budz L., Herrler G. (2001). Proteolytic activation of respiratory syncytial virus fusion protein. Cleavage at two furin consensus sequences. J. Biol. Chem..

[57] Malone B., Urakova N., Snijder E.J., Campbell E.A. (2022). Structures and functions of coronavirus replication-transcription complexes and their relevance for SARS-CoV-2 drug design. Nat. Rev. Mol. Cell Biol..

[58] Jackson C.B., Farzan M., Chen B., Choe H. (2022). Mechanisms of SARS-CoV-2 entry into cells. Nat. Rev. Mol. Cell Biol..

[59] Schultze J.L., Aschenbrenner A.C. (2021). COVID-19 and the human innate immune system. Cell.

[60] Gu Y., Zuo X., Zhang S., Ouyang Z., Jiang S., Wang F., Wang G. (2021). The Mechanism behind Influenza Virus Cytokine Storm. Viruses.

[61] Park J.H., Lee H.K. (2021). Delivery Routes for COVID-19 Vaccines. Vaccines.

[62] Flament H., Rouland M., Beaudoin L., Toubal A., Bertrand L., Lebourgeois S., Rousseau C., Soulard P., Gouda Z., Cagninacci L. (2021). Outcome of SARS-CoV-2 infection is linked to MAIT cell activation and cytotoxicity. Nat. Immunol..

[63] Carvajal J.J., Avellaneda A.M., Salazar-Ardiles C., Maya J.E., Kalergis A.M., Lay M.K. (2019). Host Components Contributing to Respiratory Syncytial Virus Pathogenesis. Front. Immunol..

[64] Short K.R., Kroeze E., Fouchier R.A.M., Kuiken T. (2014). Pathogenesis of influenza-induced acute respiratory distress syndrome. Lancet Infect. Dis..

[65] Xu Z., Shi L., Wang Y., Zhang J., Huang L., Zhang C., Liu S., Zhao P., Liu H., Zhu L. (2020). Pathological findings of COVID-19 associated with acute respiratory distress syndrome. Lancet Respir. Med..

[66] Jackson L.A., Gaglani M.J., Keyserling H.L., Balser J., Bouveret N., Fries L., Treanor J.J. (2010). Safety, efficacy, and immunogenicity of an inactivated influenza vaccine in healthy adults: A randomized, placebo-controlled trial over two influenza seasons. BMC Infect. Dis..

[67] Gao Q., Bao L., Mao H., Wang L., Xu K., Yang M., Li Y., Zhu L., Wang N., Lv Z. (2020). Development of an inactivated vaccine candidate for SARS-CoV-2. Science.

[68] Wang H., Zhang Y., Huang B., Deng W., Quan Y., Wang W., Xu W., Zhao Y., Li N., Zhang J. (2020). Development of an Inactivated Vaccine Candidate, BBIBP-CorV, with Potent Protection against SARS-CoV-2. Cell.

[69] Ghattas M., Dwivedi G., Lavertu M., Alameh M.-G. (2021). Vaccine Technologies and Platforms for Infectious Diseases: Current Progress, Challenges, and Opportunities. Vaccines.

[70] Nichol K.L., Mendelman P.M., Mallon K.P., Jackson L.A., Gorse G.J., Belshe R.B., Glezen W.P., Wittes J. (1999). Effectiveness of live, attenuated intranasal influenza virus vaccine in healthy, working adults: A randomized controlled trial. JAMA.

[71] Okamura S., Ebina H. (2021). Could live attenuated vaccines better control COVID-19?. Vaccine.

[72] Karron R.A., Buchholz U.J., Collins P.L. (2013). Live-attenuated respiratory syncytial virus vaccines. Curr. Top. Microbiol. Immunol..

[73] Caspard H., Steffey A., Mallory R.M., Ambrose C.S. (2018). Evaluation of the safety of live attenuated influenza vaccine (LAIV) in children and adolescents with asthma and high-risk conditions: A population-based prospective cohort study conducted in England with the Clinical Practice Research Datalink. BMJ Open.

[74] Rudenko L., Yeolekar L., Kiseleva I., Isakova-Sivak I. (2016). Development and approval of live attenuated influenza vaccines based on Russian master donor viruses: Process challenges and success stories. Vaccine.

[75] McFarland E.J., Karron R.A., Muresan P., Cunningham C.K., Perlowski C., Libous J., Oliva J., Jean-Philippe P., Moye J., Schappell E. (2020). Live-Attenuated Respiratory Syncytial Virus Vaccine With M2-2 Deletion and With Small Hydrophobic Noncoding Region Is Highly Immunogenic in Children. J. Infect. Dis..

[76] Karron R.A., Luongo C., Thumar B., Loehr K.M., Englund J.A., Collins P.L., Buchholz U.J. (2015). A gene deletion that up-regulates viral gene expression yields an attenuated RSV vaccine with improved antibody responses in children. Sci. Transl. Med..

[77] Rezaee F., Linfield D.T., Harford T.J., Piedimonte G. (2017). Ongoing developments in RSV prophylaxis: A clinician’s analysis. Curr. Opin. Virol..

[78] Wang Y., Yang C., Song Y., Coleman J.R., Stawowczyk M., Tafrova J., Tasker S., Boltz D., Baker R., Garcia L. (2021). Scalable live-attenuated SARS-CoV-2 vaccine candidate demonstrates preclinical safety and efficacy. Proc. Natl. Acad. Sci. USA.

[79] Xu K., Dai L., Gao G.F. (2021). Humoral and cellular immunity and the safety of COVID-19 vaccines: A summary of data published by 21 May 2021. Int. Immunol..

[80] Tioni M.F., Jordan R., Pena A.S., Garg A., Wu D., Phan S.I., Weiss C.M., Cheng X., Greenhouse J., Orekov T. (2022). Mucosal administration of a live attenuated recombinant COVID-19 vaccine protects nonhuman primates from SARS-CoV-2. NPJ Vaccines.

[81] Vetter V., Denizer G., Friedland L.R., Krishnan J., Shapiro M. (2018). Understanding modern-day vaccines: What you need to know. Ann. Med..

[82] Scorza F.B., Pardi N. (2018). New Kids on the Block: RNA-Based Influenza Virus Vaccines. Vaccines.

[83] Reina J. (2023). The new generation of messenger RNA (mRNA) vaccines against influenza. Enferm. Infecc. Microbiol. Clin..

[84] Reichmuth A.M., Oberli M.A., Jaklenec A., Langer R., Blankschtein D. (2016). mRNA vaccine delivery using lipid nanoparticles. Ther. Deliv..

[85] Echaide M., Chocarro de Erauso L., Bocanegra A., Blanco E., Kochan G., Escors D. (2023). mRNA Vaccines against SARS-CoV-2: Advantages and Caveats. Int. J. Mol. Sci..

[86] Walsh E.E., Frenck R.W., Falsey A.R., Kitchin N., Absalon J., Gurtman A., Lockhart S., Neuzil K., Mulligan M.J., Bailey R. (2020). Safety and Immunogenicity of Two RNA-Based Covid-19 Vaccine Candidates. N. Engl. J. Med..

[87] Wilson E., Goswami J., Baqui A.H., Doreski P.A., Perez-Marc G., Zaman K., Monroy J., Duncan C.J.A., Ujiie M., Ramet M. (2023). Efficacy and Safety of an mRNA-Based RSV PreF Vaccine in Older Adults. N. Engl. J. Med..

[88] Dolgin E. (2021). mRNA flu shots move into trials. Nat. Rev. Drug Discov..

[89] Abbasi J. (2021). Moderna’s mRNA Vaccine for Seasonal Flu Enters Clinical Trials. JAMA.

[90] Abbasi J. (2021). Pfizer Launches Phase 1 mRNA Flu Vaccine Trial. JAMA.

[91] Arevalo C.P., Bolton M.J., Le Sage V., Ye N., Furey C., Muramatsu H., Alameh M.G., Pardi N., Drapeau E.M., Parkhouse K. (2022). A multivalent nucleoside-modified mRNA vaccine against all known influenza virus subtypes. Science.

[92] Pardi N., Carreno J.M., O’Dell G., Tan J., Bajusz C., Muramatsu H., Rijnink W., Strohmeier S., Loganathan M., Bielak D. (2022). Development of a pentavalent broadly protective nucleoside-modified mRNA vaccine against influenza B viruses. Nat. Commun..

[93] Chapin-Bardales J., Gee J., Myers T. (2021). Reactogenicity Following Receipt of mRNA-Based COVID-19 Vaccines. JAMA.

[94] Mevorach D., Anis E., Cedar N., Bromberg M., Haas E.J., Nadir E., Olsha-Castell S., Arad D., Hasin T., Levi N. (2021). Myocarditis after BNT162b2 mRNA Vaccine against Covid-19 in Israel. N. Engl. J. Med..

[95] Heymans S., Cooper L.T. (2022). Myocarditis after COVID-19 mRNA vaccination: Clinical observations and potential mechanisms. Nat. Rev. Cardiol..

[96] Buoninfante A., Andeweg A., Genov G., Cavaleri M. (2024). Myocarditis associated with COVID-19 vaccination. NPJ Vaccines.

[97] Patone M., Mei X.W., Handunnetthi L., Dixon S., Zaccardi F., Shankar-Hari M., Watkinson P., Khunti K., Harnden A., Coupland C.A.C. (2022). Risks of myocarditis, pericarditis, and cardiac arrhythmias associated with COVID-19 vaccination or SARS-CoV-2 infection. Nat. Med..

[98] Heidary M., Kaviar V.H., Shirani M., Ghanavati R., Motahar M., Sholeh M., Ghahramanpour H., Khoshnood S. (2022). A Comprehensive Review of the Protein Subunit Vaccines Against COVID-19. Front. Microbiol..

[99] Sales V., Wang E.E. (2003). Respiratory syncytial virus vaccine: Is it coming?. Paediatr. Child. Health.

[100] Chen J., Wang J., Zhang J., Ly H. (2021). Advances in Development and Application of Influenza Vaccines. Front. Immunol..

[101] Dong Y., Dai T., Wei Y., Zhang L., Zheng M., Zhou F. (2020). A systematic review of SARS-CoV-2 vaccine candidates. Signal Transduct. Target. Ther..

[102] Treanor J. (2004). Influenza vaccine—Outmaneuvering antigenic shift and drift. N. Engl. J. Med..

[103] Gurunathan S., Klinman D.M., Seder R.A. (2000). DNA vaccines: Immunology, application, and optimization. Annu. Rev. Immunol..

[104] Momin T., Kansagra K., Patel H., Sharma S., Sharma B., Patel J., Mittal R., Sanmukhani J., Maithal K., Dey A. (2021). Safety and Immunogenicity of a DNA SARS-CoV-2 vaccine (ZyCoV-D): Results of an open-label, non-randomized phase I part of phase I/II clinical study by intradermal route in healthy subjects in India. EClinicalMedicine.

[105] Khobragade A., Bhate S., Ramaiah V., Deshpande S., Giri K., Phophle H., Supe P., Godara I., Revanna R., Nagarkar R. (2022). Efficacy, safety, and immunogenicity of the DNA SARS-CoV-2 vaccine (ZyCoV-D): The interim efficacy results of a phase 3, randomised, double-blind, placebo-controlled study in India. Lancet.

[106] Smith T.R.F., Patel A., Ramos S., Elwood D., Zhu X., Yan J., Gary E.N., Walker S.N., Schultheis K., Purwar M. (2020). Immunogenicity of a DNA vaccine candidate for COVID-19. Nat. Commun..

[107] Tebas P., Yang S., Boyer J.D., Reuschel E.L., Patel A., Christensen-Quick A., Andrade V.M., Morrow M.P., Kraynyak K., Agnes J. (2021). Safety and immunogenicity of INO-4800 DNA vaccine against SARS-CoV-2: A preliminary report of an open-label, Phase 1 clinical trial. EClinicalMedicine.

[108] Kraynyak K.A., Blackwood E., Agnes J., Tebas P., Giffear M., Amante D., Reuschel E.L., Purwar M., Christensen-Quick A., Liu N. (2022). SARS-CoV-2 DNA Vaccine INO-4800 Induces Durable Immune Responses Capable of Being Boosted in a Phase 1 Open-Label Trial. J. Infect. Dis..

[109] Tatsis N., Ertl H.C. (2004). Adenoviruses as vaccine vectors. Mol. Ther..

[110] Vanaparthy R., Mohan G., Vasireddy D., Atluri P. (2021). Review of COVID-19 viral vector-based vaccines and COVID-19 variants. Infez. Med..

[111] Travieso T., Li J., Mahesh S., Mello J., Blasi M. (2022). The use of viral vectors in vaccine development. NPJ Vaccines.

[112] Matsuda K., Migueles S.A., Huang J., Bolkhovitinov L., Stuccio S., Griesman T., Pullano A.A., Kang B.H., Ishida E., Zimmerman M. (2021). A replication-competent adenovirus-vectored influenza vaccine induces durable systemic and mucosal immunity. J. Clin. Investig..

[113] Sayedahmed E.E., Elkashif A., Alhashimi M., Sambhara S., Mittal S.K. (2020). Adenoviral Vector-Based Vaccine Platforms for Developing the Next Generation of Influenza Vaccines. Vaccines.

[114] Butler C., Ellis C., Folegatti P.M., Swayze H., Allen J., Bussey L., Bellamy D., Lawrie A., Eagling-Vose E., Yu L.M. (2021). Efficacy and Safety of a Modified Vaccinia Ankara-NP+M1 Vaccine Combined with QIV in People Aged 65 and Older: A Randomised Controlled Clinical Trial (INVICTUS). Vaccines.

[115] Diez-Domingo J., Saez-Llorens X., Rodriguez-Weber M.A., Epalza C., Chatterjee A., Chiu C.H., Lin C.Y., Berry A.A., Martinon-Torres F., Baquero-Artigao F. (2023). Safety and Immunogenicity of a ChAd155-Vectored Respiratory Syncytial Virus (RSV) Vaccine in Healthy RSV-Seropositive Children 12-23 Months of Age. J. Infect. Dis..

[116] Jordan E., Kabir G., Schultz S., Silbernagl G., Schmidt D., Jenkins V.A., Weidenthaler H., Stroukova D., Martin B.K., De Moerlooze L. (2023). Reduced Respiratory Syncytial Virus Load, Symptoms, and Infections: A Human Challenge Trial of MVA-BN-RSV Vaccine. J. Infect. Dis..

[117] Falsey A.R., Hosman T., Bastian A.R., Vandenberghe S., Chan E.K.H., Douoguih M., Heijnen E., Comeaux C.A., Callendret B., on behalf of the CYPRESS Investigators (2024). Long-term efficacy and immunogenicity of Ad26.RSV.preF-RSV preF protein vaccine (CYPRESS): A randomised, double-blind, placebo-controlled, phase 2b study. Lancet Infect. Dis..

[118] Mendonca S.A., Lorincz R., Boucher P., Curiel D.T. (2021). Adenoviral vector vaccine platforms in the SARS-CoV-2 pandemic. NPJ Vaccines.

[119] Zhao T., Cai Y., Jiang Y., He X., Wei Y., Yu Y., Tian X. (2023). Vaccine adjuvants: Mechanisms and platforms. Signal Transduct. Target. Ther..

[120] Coffman R.L., Sher A., Seder R.A. (2010). Vaccine adjuvants: Putting innate immunity to work. Immunity.

[121] Bode C., Zhao G., Steinhagen F., Kinjo T., Klinman D.M. (2011). CpG DNA as a vaccine adjuvant. Expert. Rev. Vaccines.

[122] Verma S.K., Mahajan P., Singh N.K., Gupta A., Aggarwal R., Rappuoli R., Johri A.K. (2023). New-age vaccine adjuvants, their development, and future perspective. Front. Immunol..

[123] Turley J.L., Lavelle E.C. (2022). Resolving adjuvant mode of action to enhance vaccine efficacy. Curr. Opin. Immunol..

[124] O’Hagan D.T., van der Most R., Lodaya R.N., Coccia M., Lofano G. (2021). “World in motion”—Emulsion adjuvants rising to meet the pandemic challenges. NPJ Vaccines.

[125] Budroni S., Buricchi F., Cavallone A., Bourguignon P., Caubet M., Dewar V., D’Oro U., Finco O., Garcon N., El Idrissi M. (2021). Antibody avidity, persistence, and response to antigen recall: Comparison of vaccine adjuvants. NPJ Vaccines.

[126] Bernstein D.I., Edwards K.M., Dekker C.L., Belshe R., Talbot H.K., Graham I.L., Noah D.L., He F., Hill H. (2008). Effects of adjuvants on the safety and immunogenicity of an avian influenza H5N1 vaccine in adults. J. Infect. Dis..

[127] Wei C.J., Crank M.C., Shiver J., Graham B.S., Mascola J.R., Nabel G.J. (2020). Next-generation influenza vaccines: Opportunities and challenges. Nat. Rev. Drug Discov..

[128] Shinde V., Cho I., Plested J.S., Agrawal S., Fiske J., Cai R., Zhou H., Pham X., Zhu M., Cloney-Clark S. (2022). Comparison of the safety and immunogenicity of a novel Matrix-M-adjuvanted nanoparticle influenza vaccine with a quadrivalent seasonal influenza vaccine in older adults: A phase 3 randomised controlled trial. Lancet Infect. Dis..

[129] Stertman L., Palm A.E., Zarnegar B., Carow B., Lunderius Andersson C., Magnusson S.E., Carnrot C., Shinde V., Smith G., Glenn G. (2023). The Matrix-M adjuvant: A critical component of vaccines for the 21(st) century. Hum. Vaccin. Immunother..

[130] Lv X., Martin J., Hoover H., Joshi B., Wilkens M., Ullisch D.A., Leibold T., Juchum J.S., Revadkar S., Kalinovska B. (2024). Chemical and biological characterization of vaccine adjuvant QS-21 produced via plant cell culture. iScience.

[131] Coleman B.L., Gutmanis I., McGovern I., Haag M. (2023). Effectiveness of Cell-Based Quadrivalent Seasonal Influenza Vaccine: A Systematic Review and Meta-Analysis. Vaccines.

[132] Rockman S., Laurie K., Ong C., Rajaram S., McGovern I., Tran V., Youhanna J. (2023). Cell-Based Manufacturing Technology Increases Antigenic Match of Influenza Vaccine and Results in Improved Effectiveness. Vaccines.

[133] Sandbulte M.R., Westgeest K.B., Gao J., Xu X., Klimov A.I., Russell C.A., Burke D.F., Smith D.J., Fouchier R.A., Eichelberger M.C. (2011). Discordant antigenic drift of neuraminidase and hemagglutinin in H1N1 and H3N2 influenza viruses. Proc. Natl. Acad. Sci. USA.

[134] Chavda V.P., Bezbaruah R., Deka K., Nongrang L., Kalita T. (2022). The Delta and Omicron Variants of SARS-CoV-2: What We Know So Far. Vaccines.

[135] Zimmerman T., Shiroma K., Fleischmann K.R., Xie B., Jia C., Verma N., Lee M.K. (2023). Misinformation and COVID-19 vaccine hesitancy. Vaccine.

[136] Russell M.W., Moldoveanu Z., Ogra P.L., Mestecky J. (2020). Mucosal Immunity in COVID-19: A Neglected but Critical Aspect of SARS-CoV-2 Infection. Front. Immunol..

[137] Hassan A.O., Kafai N.M., Dmitriev I.P., Fox J.M., Smith B.K., Harvey I.B., Chen R.E., Winkler E.S., Wessel A.W., Case J.B. (2020). A Single-Dose Intranasal ChAd Vaccine Protects Upper and Lower Respiratory Tracts against SARS-CoV-2. Cell.

[138] Bricker T.L., Darling T.L., Hassan A.O., Harastani H.H., Soung A., Jiang X., Dai Y.N., Zhao H., Adams L.J., Holtzman M.J. (2021). A single intranasal or intramuscular immunization with chimpanzee adenovirus-vectored SARS-CoV-2 vaccine protects against pneumonia in hamsters. Cell Rep..

[139] Hassan A.O., Feldmann F., Zhao H., Curiel D.T., Okumura A., Tang-Huau T.L., Case J.B., Meade-White K., Callison J., Chen R.E. (2021). A single intranasal dose of chimpanzee adenovirus-vectored vaccine protects against SARS-CoV-2 infection in rhesus macaques. Cell Rep. Med..

[140] Mettelman R.C., Allen E.K., Thomas P.G. (2022). Mucosal immune responses to infection and vaccination in the respiratory tract. Immunity.

[141] Dotiwala F., Upadhyay A.K. (2023). Next Generation Mucosal Vaccine Strategy for Respiratory Pathogens. Vaccines.

[142] Murphy B.R., Clements M.L. (1989). The systemic and mucosal immune response of humans to influenza A virus. Curr. Top. Microbiol. Immunol..

[143] Neutra M.R., Kozlowski P.A. (2006). Mucosal vaccines: The promise and the challenge. Nat. Rev. Immunol..

[144] Mayer L., Shao L. (2004). Therapeutic potential of oral tolerance. Nat. Rev. Immunol..

[145] Tang R., Zheng H., Wang B.S., Gou J.B., Guo X.L., Chen X.Q., Chen Y., Wu S.P., Zhong J., Pan H.X. (2023). Safety and immunogenicity of aerosolised Ad5-nCoV, intramuscular Ad5-nCoV, or inactivated COVID-19 vaccine CoronaVac given as the second booster following three doses of CoronaVac: A multicentre, open-label, phase 4, randomised trial. Lancet Respir. Med..

[146] Singh C., Verma S., Reddy P., Diamond M.S., Curiel D.T., Patel C., Jain M.K., Redkar S.V., Bhate A.S., Gundappa V. (2023). Phase III Pivotal comparative clinical trial of intranasal (iNCOVACC) and intramuscular COVID 19 vaccine (Covaxin((R))). NPJ Vaccines.

[147] Jeyanathan M., Afkhami S., Kang A., Xing Z. (2023). Viral-vectored respiratory mucosal vaccine strategies. Curr. Opin. Immunol..

